# Influence of uncertainty on framed decision-making with moral dilemma

**DOI:** 10.1371/journal.pone.0197923

**Published:** 2018-05-30

**Authors:** Gaëtan Merlhiot, Martial Mermillod, Jean-Luc Le Pennec, Frédéric Dutheil, Laurie Mondillon

**Affiliations:** 1 Université Clermont Auvergne, CNRS, LAPSCO, Physiological and Psychosocial Stress, LABEX ClerVolc, Clermont-Ferrand, France; 2 Univ. Grenoble Alpes, LPNC & CNRS, UMR 5105 LPNC, Grenoble, France; 3 Univ. Grenoble Alpes, LJK & CNRS, UMR 5524 LJK, Grenoble, France; 4 Université Clermont Auvergne, CNRS, IRD, OPGC, Laboratoire Magmas et Volcans, LABEX ClerVolc, Clermont-Ferrand, France; 5 Université Clermont Auvergne, CNRS, LAPSCO, Stress physiologique et psychosocial, CHU Clermont-Ferrand, Santé Travail Environnement, WittyFit, Clermont-Ferrand, France; 6 Australian Catholic University, Faculty of Health, Melbourne, Victoria, Australia; Southwest University, CHINA

## Abstract

In cases of impending natural disasters, most events are uncertain and emotionally relevant, both critical factors for decision-making. Moreover, for exposed individuals, the sensitivity to the framing of the consequences (gain or loss) and the moral judgments they have to perform (e.g., evacuate or help an injured person) constitute two central effects that have never been examined in the same context of decision-making. In a framed decision-making task with moral dilemma, we investigated whether uncertainty (i.e., unpredictably of events) and a threatening context would influence the framing effect (actions framed in loss are avoided in comparison to the ones framed in gain) and the personal intention effect (unintentional actions are more morally acceptable in comparison to intentional actions) on the perceived moral acceptability of taking action. Considering the impact of uncertainty and fear on the processes underlying these effects, we assumed that these emotions would lead to the negation of the two effects. Our results indicate that the exposure to uncertain events leads to the negation of the framing effect, but does not influence the moral acceptability and the effect of personal intention. We discuss our results in the light of dual-process models (i.e. systematic *vs*. heuristic), appraisal theories, and neurocognitive aspects. These elements highlight the importance of providing solutions to cope with uncertainty, both for scientists and local populations exposed to natural hazards.

## Introduction

Natural disasters present recurrent uncertain and unpredictable events, which are constantly dealt with by scientists and populations at risk [1,2] and systematically lead to states of fear and anxiety [3]. Because uncertainty (defined as the low predictability of events or outcomes) and threatening context are known to alter decision-making (for reviews: [4,5]), we considered their influences on two effects that modulate decision-making associated with a moral dilemma [6]: the framing effect [7,8] and the effect of personal intention [9].

### Moral decision-making and emotions

When decisions are made in a situation of imminent threat, moral dilemmas, including both emotional and rational aspects [6,10], are often a source of complication. For example, the *Footbridge dilemma* [11] involves an out-of-control trolley that is about to kill five people if not stopped. Two options are available: *(i)* do nothing (moral response) or *(ii)* accept a five-for-one tradeoff that corresponds to stopping the trolley by pushing a pedestrian over the track, killing him to save these five people (utilitarian response). Incidental emotions (feelings or moods felt before and during a decision, not fundamentally related to the judgment or decision [5,12,13]) could influence moral judgments in this dilemma. For example, positive emotions induced by humorous video would decrease moral responses [14]. However, it appeared that some positive emotions (mirth) decreased moral responses whereas others (elevation) increased them [15]. Moreover, utilitarian responses would decrease with happiness and increase with sadness when focusing on the action, but the opposite pattern would be found when focusing on the non-action [16]. To explain these discrepancies, some authors have proposed that incidental emotions could change how the dilemmas are appraised and the resulting anticipatory emotion [14,15] inherent to the dilemma (emotions arising from the anticipation/visualization of the outcomes [5,12,13]).

Greene et al. [10] introduced a dual-process theory (for a review: [17]) to account for the effects of anticipatory emotions on moral decision-making. In this model, judging to be morally unacceptable to take action (moral response: choosing to do nothing) derives from an automatic emotional response (system 1: a fast, intuitive, automatic, parallel, and emotionally dependent process) as the negative anticipatory emotions aroused by the five-for-one tradeoff option are not inhibited. Conversely, judging it to be morally acceptable to take action (utilitarian response: choosing the five-for-one tradeoff) results from controlled rational concerns (system 2: a slow, controlled, serial, and emotionally independent process) and constitutes a cognitive cost as it requires inhibiting the anticipatory negative emotions associated with the tradeoff option [18]. But it is possible to lessen the negative anticipatory emotions aroused by the tradeoff option by removing the personal intention, that is to say the “personal intent to kill” [9,19]. For example, in another version of the footbridge dilemma, a switch can be used to drive the trolley to a sidetrack, where it still kills one person. Because the victim is not pushed, most people perceive their death as coming from an unintentional action, and thus accept the five-for-one tradeoff. Consequently, the moral acceptability of the utilitarian response would increase by reducing the processing of anticipatory emotions. Interestingly, this reduction happens when people are exposed to uncertainty and uncertainty-associated emotions [20,21].

### The central role of (un)certainty

(Un)certainty contributes to the advent of incidental emotions. For example, uncertainty often gives rise to fear [3]. However, according to the *appraisal* theories [12,22] and the *appraisal tendency framework* [23,24], uncertainty, which precedes valence appraisal [22,25], even characterizes emotions and information processing tendencies: *(i)* uncertainty-associated emotions (*e*.*g*., fear, sadness) involve increasingly systematic processes (system 2) that reduce the processing of anticipatory emotions; *(ii)* certainty-associated emotions (*e*.*g*., anger, happiness) involve increasingly heuristic processes (system 1) that strengthen the processing of anticipatory emotions. This central role of (un)certainty on the dual process model is now clearly supported by advances in neurosciences: Uncertainty, which is attributable to the occurrence of unexpected or unfamiliar events [25,26], elicits orientation responses and states of arousal and alertness [3,25]. But this arousal response, associated with an heightened activity of the cerebral amygdala [3,25,27,28], is immediately regulated by the prefrontal areas [3] and systematic processes (system 2) [4,17,30]. Consequently, increasing the certainty of an uncertainty-associated emotion by means of a high predictability of events and outcomes would limit the use of systematic processes (system 2) [20] and the reduction of the processing of anticipatory emotions [20,21]. We thus posit that (un)certainty by itself would constitute a critical factor to the changes in the decision-making process.

In addition, the salience of positive (gain) or negative (loss) aspects of the choices also drives decisions: this is called the framing effect [7]. For example, when individuals have to choose between two options with 90% chance of success or 10% risk of failure, they prefer the option with 90% chance of success, although both options are actually equivalent. The underlying mechanism of this effect is known as the *loss aversion*. It corresponds to the tendency to overweight loss toward gain and results from the negative anticipatory emotional response arising from loss, emphasized by heuristic processing (system 1) [31,32]. Interestingly, this effect has already been observed in a moral dilemma where the framing of gain, compared to the framing of loss, increased the moral acceptability of taking action [8]. For this reason, as uncertainty and uncertainty-associated emotions imply the use of systematic processes (system 2) and a reduction of the processing of anticipatory emotions arising from loss [20,21], we can expect that they would lead to the negation of the framing effect in a moral dilemma task.

### The present research

We investigated the influence of uncertainty and a threatening context stemming from unpredictable events on two effects that modulate decision-making associated with a moral dilemma: the framing effect and the effect of personal intention. Both of these can modulate the negative anticipatory emotions associated to the utilitarian response. For the purposes of the study, we designed a paradigm to elicit (un)certainty (certainty *vs*. uncertainty) responses by means of (un)predictable (unpredictable *vs*. predictable) stimuli either threatening or neutral. We used a visual presentation of the moral dilemmas (presenting the consequences of both the action and non-action), framed in terms of gain or loss and divided into two degrees of personal intentions—namely, intentional and unintentional actions.

According to the above-mentioned literature, threatening context and uncertainty (i.e., unpredictability of events) would reduce the processing of anticipatory emotions [20,21,23] leading to an increase of moral acceptability of the action [9]. Consequently, we hypothesized that a threatening context and uncertainty would lead to the negation of the framing effect [31,33] while neutrality and certainty would maintain it. We also expected that a threatening context and uncertainty would reduce the effect of personal intention [9] while neutrality and certainty would increase it. These effects were depicted in our design as follows: In the threatening context and uncertainty conditions, there would be no difference in the moral acceptability of taking action between the loss and gain framings and between the intentional and unintentional actions when compared to neutrality and certainty conditions. Finally, as anxiety is very close to the uncertainty-associated emotions and based on the fact that it is associated with arousal via an heightened amygdala response (e.g., [3,27]), we assumed that anxiety would present similar effects on the moral acceptability scores.

## Method

### Participants

We recruited 131 undergraduate students (121 females, 10 males) with a mean age of 19.71 ± 2.48 years from the Clermont Auvergne University (formerly Blaise Pascal University) in Clermont-Ferrand (France). All signed a written informed consent form and received course credits for their participation. The protocol was approved by the Ethics Committee of Clermont-Ferrand (ref: HMC_14–15, IRB00008526, 2014/CE49).

### Exclusion criterion

Participants were excluded from the study if they suffered from specific phobia(s), based on DSM-IV diagnostic criteria (N = 0) and evaluated by a psychologist (the experimenter).

### Material and design

#### Induction task

**Fig 1:**
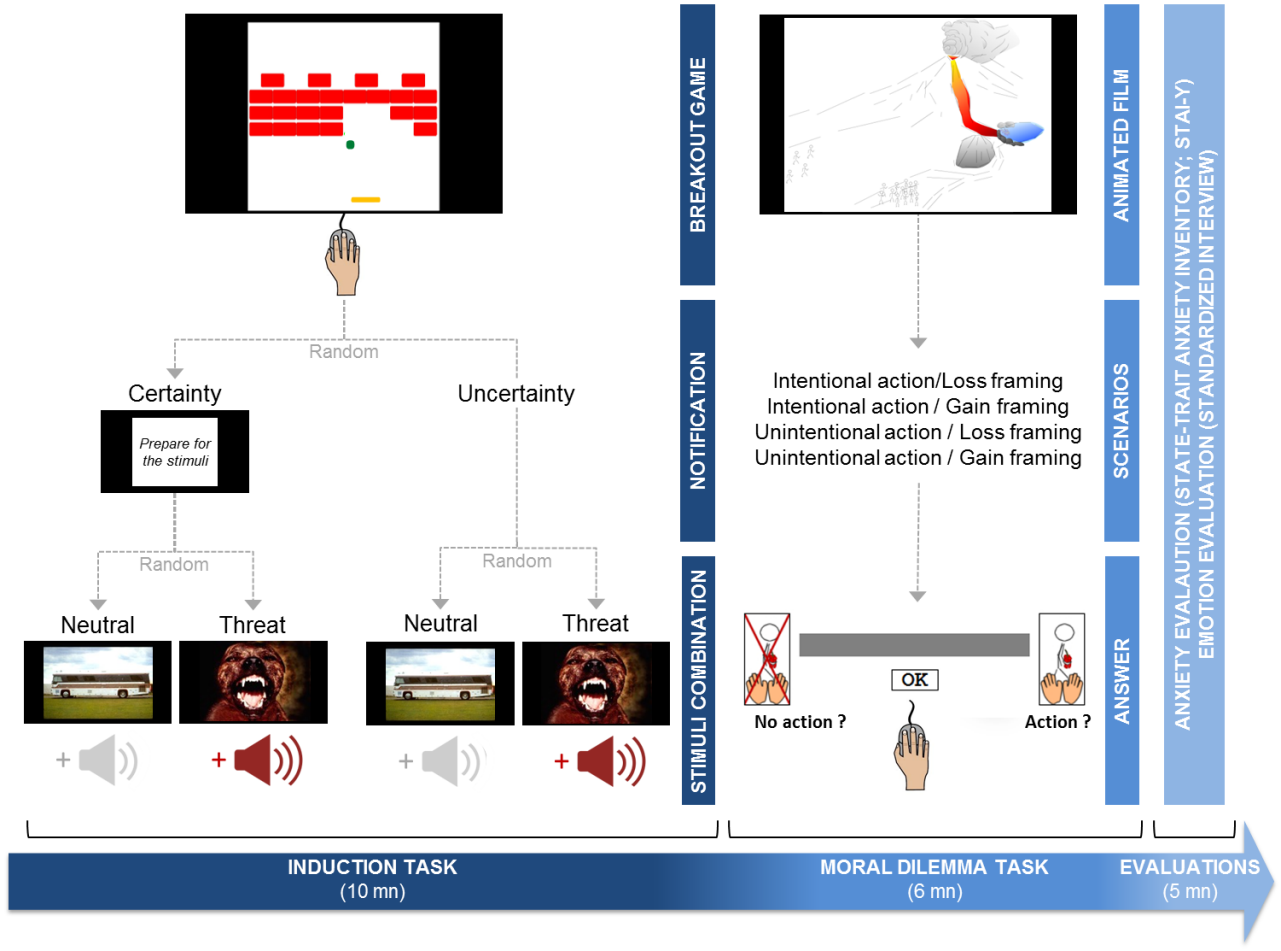
Time course of the experimental design, including the induction task, the moral dilemma task, and the anxiety and emotion evaluations.

The induction consisted of a computerized breakout game designed for the experiment. The goal of the game is to destroy different layers of bricks by means of a ball, bouncing it off the top and sides of the screen. If the ball reaches the bottom of the screen, the player loses a turn and the ball restarts from its starting position. To avoid this, the player controls a vertical paddle (with the mouse) to bounce the ball against the bricks (S1 Video). The game was randomly interrupted by the sudden presentation of auditory/visual stimuli combinations related to threat or neutrality, in a random between-subjects design (Fig 1). All stimuli were extracted from the IAPS [34] and the IADS [35] databases. The threat-associated stimuli entailed 5 aversive sounds of screams repeated once with 10 aversive pictures, and the neutral combinations involved 5 neutral sounds repeated once with 10 neutral pictures. The threatening pictures from the IAPS were: 1022 (snake), 1040 (snake), 1050 (snake), 1120 (snake), 1300 (dog), 1930 (shark), 1931 (shark), 6244 (aimed gun), 6250 (aimed gun), 6260 (aimed gun); the threatening sounds from the IADS were: 275 (scream, 0:01 to 0:03), 275 (scream, 0:035 to 0:055), 276 (female scream, 0:00 to 0:02), 277 (female scream, 0:00 to 0:02), 277 (female scream, 0:04 to 0:06). The neutral pictures from the IAPS were: 1675 (buffalo), 5395 (boat), 7036 (shipyard), 7037 (trains), 7140 (bus), 7150 (umbrella), 7184 (abstract art), 7211 (clock), 7500 (building), 7560 (freeway); the neutral sounds from the IADS were: 170 (night, 0:00 to 0:02), 322 (typewriter, 0:00 to 0:02), 376 (lawnmower, 0:00 to 0:02) 425 (train, 0:00 to 0:02), 722 (walking, 0:00 to 0:02).

Each stimuli combination appeared for 2000ms, with a random onset that varied according to two levels of predictability in a random between-subjects design. In the high predictability (certainty) condition, a notification “*Prepare for the stimuli*” appeared for 2000ms before the onset of the stimuli. In the condition of low predictability (uncertainty), there was no notification. The first stimuli could appear within a time frame of 10s to 50s, and the second, up to the tenth stimuli, could appear after 20 to 60s from the end of the first stimuli period. This allowed a pseudo-random distribution over a 10-minute timespan for the induction period.

#### Moral dilemma task

**Fig 2:**
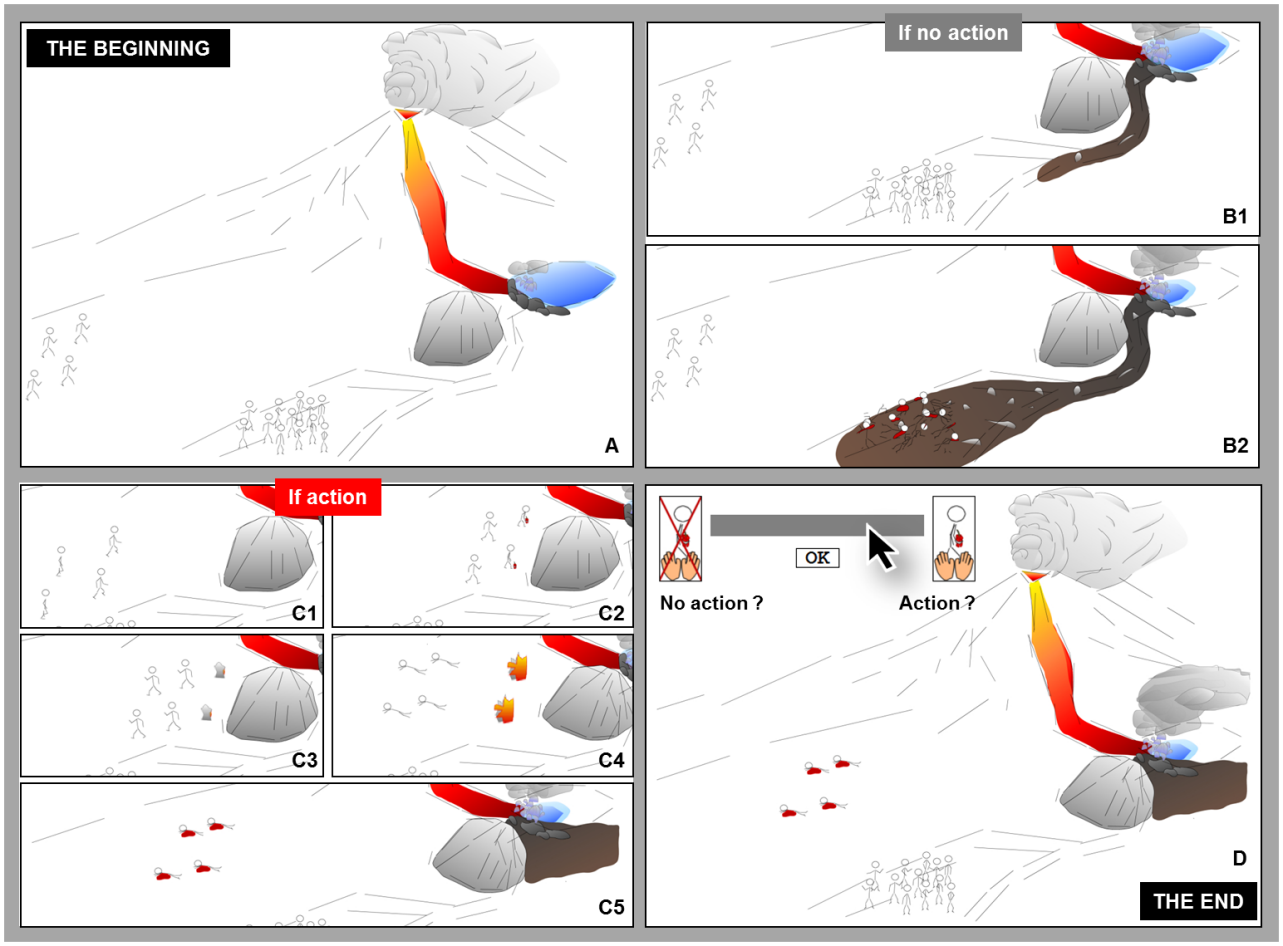
Examples of the progress of the animated intentional scenarios for the loss frame effect, where some individuals are sent to their inescapable fate by dynamiting a rocky pan to stop the progress of a lahar. Notes: **(A)** a lava flow is about to collapse the lake; If no action: **(B1)** the collapsed lake would release a lahar and **(B2)** kill the inhabitants; If action: **(C1)** some individuals can be sent to a rocky pan and **(C2)** use dynamite to divert the course of the lahar, but **(C3,C4)** the individuals won’t be able to flee from the explosion and **(C5)** they would be killed by the explosion to save the other inhabitants; **(D)** participants have to use the grey bar to give their answer.

For the task, which was inspired by the paradigm of the Footbridge trolley dilemma [11], we manipulated the participant’s personal intention (intentional or unintentional action) and the framing of the outcome (gain or loss), resulting in four scenarios in a fully random within-subjects design (Fig 2) (S2 Video). In all scenarios, which were in the form of an animated film, a lahar (type of mudslide flowing down from a volcano), resulting of a lava flow that entered a volcanic lake and collapsed the inside structure of the lake, threatened the inhabitants of a valley. The task consisted of choosing between two options: *i*) to do nothing and to let the lahar progress and kill all the inhabitants (no action), or *ii*) to stop its progress by dynamiting a rocky pan which would cause some victims by its fall (action). We controlled the number of exposed people while keeping the same ratio of survivor/casualties (1/3, 2/6, 4/12), in order to obtain 12 different trials.

*Manipulation of the personal intention*. Following the example of a previous variant of the trolley dilemma [9], in which the personal intention was already manipulated, we created two scenarios. In the “intentional action” scenario, it was possible to dynamite the rocky pan by summoning people without telling them that they would inevitably die from the explosion (intentional death caused by side-effect). In the “unintentional action” scenario, it was possible to dynamite the rocky pan by pressing a button, which would also involve the death of people around (unintentional death caused by side-effect).

*Manipulation of the framing*. We used a visual variant of a previous version of the paradigm framed in terms of gain or loss [7,36]. For this purpose, we created two additional animations: survivors jumping for joy for the gain framing, and a pool of blood flowing from the dead bodies for the loss framing. We specified that, since the scenarios describing the dilemma were identical in both conditions (gain and loss), all participants were aware that some individuals would die and some others would survive, depending on the choices (“no action” and “action”). Each trial began with a short scene depicting the scenario. Then the consequences of the “no action” and “action” choices were presented. Finally, the participant had to answer by clicking on a continuous bar ranging from “no action” to “action”. Participants had 30 s to give their answer before an alert message was emitted requesting him/her to give an answer as quickly as possible. The moral acceptability (of taking action) score varied from 0% (to judge it immoral to take action) to 100% (to judge it moral to take action). The response-time (RT) for each trial was also recorded in ms.

#### Other evaluations

*Anxiety evaluation*. We used the French version of the State-Trait Anxiety Inventory (STAI-Y) [37,38]. Each subscale consisted of 20 items scored on a 4-point Likert scale, with the score varying from 20 to 80. A high score corresponded to a high level of state- and trait-anxiety.

*Emotion evaluation*. We used a standardized interview to assess which emotion the participant felt during the induction task. The questions are summarized below: *“During and/or after the completion of the first task (the breakout game) have you felt any emotion*, *positive or negative*?*–If the answer was yes*, *can you describe those feelings*?*–Did you feel any anxiety or stress*? *Did you feel any excitement*, *tenseness or arousal*?*”* To ensure veracity of the answers, any answer was followed by a confirmation question. In order to get the highest specificity, only the presence of the emotion was taken into account and not its intensity.

### Procedure

The experimenter used standardized instructions and verified the presence of any exclusion criterion (*e*.*g*., dog or snake phobia). Each participant was randomized into one of the four induction conditions: certainty/threatening, uncertainty/threatening, certainty/neutral, uncertainty/neutral. The experimenter then left the participant alone for the entire length of the protocol. All participants first performed the induction task (10 min) (S1 Video), then the moral dilemma task where they were instructed to give spontaneous answers (about 6 min) (S2 Video), and finally they completed the STAI-Y task (about 5 min). The tasks ran under E-Prime 2.0 (Psychology Software Tools, Pittsburg, PA) on a PC with a 17-inche screen (4:3). The entire protocol lasted about 20 min. Finally, the experimenter conducted the emotion evaluation using the standardized interview (about 5 min).

### Statistical analysis

The framing effect was the main outcome. We estimated the effect size reported in the literature (e.g., ref. [33,39,40]), which appears to be relatively strong (η^2^ = .3 ± .1, Cohen’s d = .7 ± .1). We therefore conducted a power analysis using Gpower [41], which indicated that a sample size of minimum 72 people would be required to detect a framing effect with an effect size of η^2^ = .3 using a repeated ANOVA. We excluded participants with an absence of fear/anxiety and/or a presence of happiness/excitement following the induction of a threatening context (N = 6): feelings of happiness while watching and hearing strong aversive stimuli were considered as aberrant and abnormal responses, suggesting those participants did not perform the task properly; no participant reported the presence of emotions following the induction of a neutral context. We also excluded those who did not complete the whole task (N = 5): two participants did not perform the breakout game and three declared that they did not complete the moral dilemma task correctly as they intentionally gave the same answers for all trials. The final sample size was 120 participants (age of 19.68 ± 2.51; male/female ratio: 9/111) randomly assigned to one of the four experimental groups: certainty/threatening (age = 20.63 ± 4.15; ratio = 3/27), uncertainty/threatening (age = 19.03 ± 1.1; ratio = 2/28), certainty/neutral (age = 19.33 ± 1.81; ratio = 0/30), and uncertainty/neutral (age = 19.73 ± 1.62; ratio = 4/26).

Tests were two-sided, with a type I error set at α = 0.05. Data are presented as mean percentage change ± SD. The assumption of Gaussian distribution for each parameter was assessed using a Kolmogorov-Smirnov test. The Kolmogorov-Smirnov test stratified for context (threatening *vs*. neutral), (un)certainty (certainty *vs*. uncertainty), framing (gain, loss), and personal intention (intentional, unintentional) conditions showed that the moral acceptability scores satisfied the requirements for normality (*p* > .05). The RTs presented a high shrewdness that was reduced using the reciprocal RT (-1/RT), a reliable transformation for the use of statistical analysis based on normal-distribution [42].

We first conducted a repeated-measures ANOVA on the moral acceptability scores and RT, with context (threatening *vs*. neutral) and (un)certainty (certainty *vs*. uncertainty) as a between-subjects variable, and with framing (gain, loss) and personal intention (intentional, unintentional) as a within-subjects variable. Levene’s test established that the equality of variance was respected (*p* > .05). Significant interactions were followed by simple effect analyses with Bonferroni corrections. As complementary information, we compared the results of the repeated-measures ANOVA with a LMM, with subjects and within-subject conditions as random effects, and we obtained strictly identical results.

Finally, we examined whether state anxiety predicted the moral acceptability score as a function of the framing (gain, loss) and the personal intention (intentional, unintentional) effects. We conducted ANOVAs on state and trait anxiety scores with context (threatening *vs*. neutral) and (un)certainty (certainty *vs*. uncertainty) as the between-subjects variable. The Levene’s test established that the equality of variance was respected (p > .05). We then computed two difference scores: the difference in the moral acceptability between the framings (loss—gain), and the difference between the personal intentions (intentional—unintentional actions). We regressed those difference scores on both the state-anxiety and the trait-anxiety scores.

Statistical analyses were conducted using IBM SPSS 22 (IBM Corp., USA). The significance level was set at *p* ≤ .05, and the trend level was set at *p* ≤ .07.

## Results

All results (all main effects and interactions, all means and standard deviations) of the repeated-measures ANOVA on the moral acceptability score are available as supplementary material (S1 Table).

We obtained a main effect of the framing (gain, loss) on the moral acceptability score, where participants in the loss condition presented lower scores (*M* = 47.97, *SD* = 22.56) than in the gain condition (*M* = 51.89, *SD* = 22.15), *F*(1, 116) = 11.93, *p* = .001, η_p_^2^ = .093. We also found a main effect of the personal intention (intentional, unintentional), where unintentional actions resulted in higher scores (*M* = 54.09, *SD* = 22.58) than intentional actions (*M* = 45.76, *SD* = 23.58), *F*(1, 116) = 27.63, *p* < .001, η_p_^2^ = .19. We obtained a marginal effect of the context (threatening *vs*. neutral) on the moral acceptability scores, where participants in the threatening context condition presented higher scores (*M* = 53.56, *SD* = 21.42) than participants in the neutral context condition (*M* = 46.30, *SD* = 20.94), *F*(1, 116) = 3.49, *p* = .064, η_p_^2^ = .029. There was no main effect of (un)certainty (certainty *vs*. uncertainty), *F*(1, 116) = 0.017, *p* = .896, η_p_^2^ = .0002.

**Fig 3:**
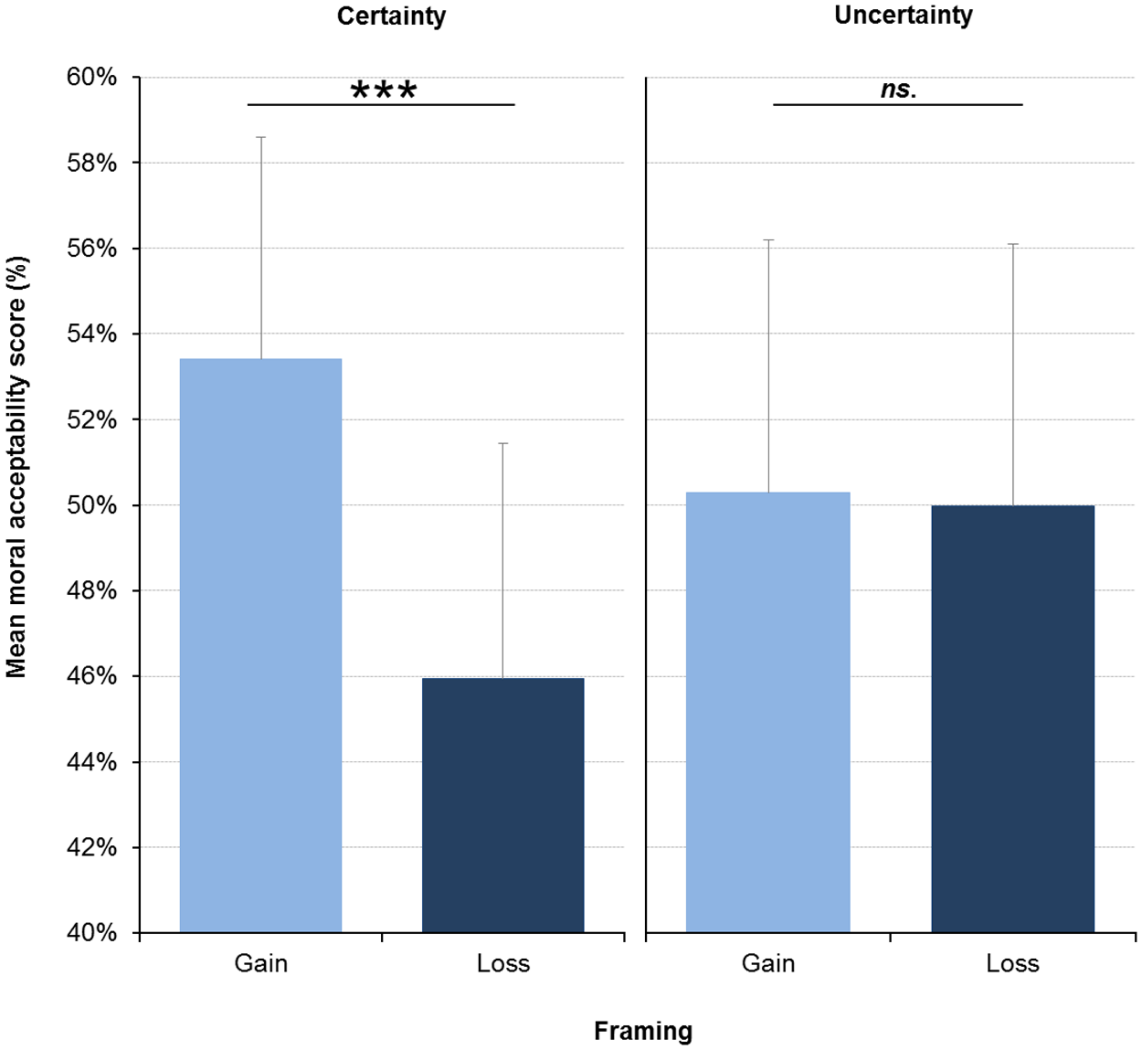
Mean moral acceptability score as a function of framing (gain, loss) and (un)certainty (certainty *vs*. uncertainty). Bars represent 95% CI.

The results showed an interaction effect between the framing (gain, loss) and the (un)certainty (certainty *vs*. uncertainty), *F*(1, 116) = 9.62, *p* = .002, η_p_^2^ = .077 (Fig 3). In the certainty condition, the framing of gain involved higher moral acceptability scores (*M* = 53.40, *SD* = 21.45) than the framing of loss (*M* = 45.95, *SD* = 21.35), *F*(1, 116) = 21.48, *p* < .001, η_p_^2^ = .16. However, there was no difference between framings of gain and loss in the uncertainty condition, *F*(1, 116) = 0.062, *p* = .804, η_p_^2^ = .001. In addition, no significant differences were found between the certainty and uncertainty conditions within the framing of gain, *F*(1, 116) = 0.57, *p* = .453, η_p_^2^ = .005, or within the framing of loss, *F*(1, 116) = 0.97, *p* = .326, η_p_^2^ = .008. The interaction effect between the personal intention (intentional, unintentional) and the (un)certainty (certainty *vs*. uncertainty) was not significant, *F*(1, 116) = 0.780, *p* = .379, η_p_^2^ = .007. The interaction effects between the framing (gain, loss) and the context (threatening *vs*. neutral), and between the personal intention (intentional, unintentional) and the context (threatening *vs*. neutral) were not significant, *F*(1, 116) = 1.07, *p* = .3 and *F*(1, 116) = .02, *p* = .89 respectively.

The RT analysis revealed only a main effect of the (un)certainty (certainty *vs*. uncertainty) condition, with the participants in the uncertainty condition presenting longer RT (*M* = 5328.27, *SD* = 1580.14) compared to participants in the certainty condition (*M* = 6153.55, *SD* = 2240.63), *F*(1, 116) = 4.06, *p* = .046, η_p_^2^ = .03. All other effects were non-significant (*p* > .15)

**Fig 4:**
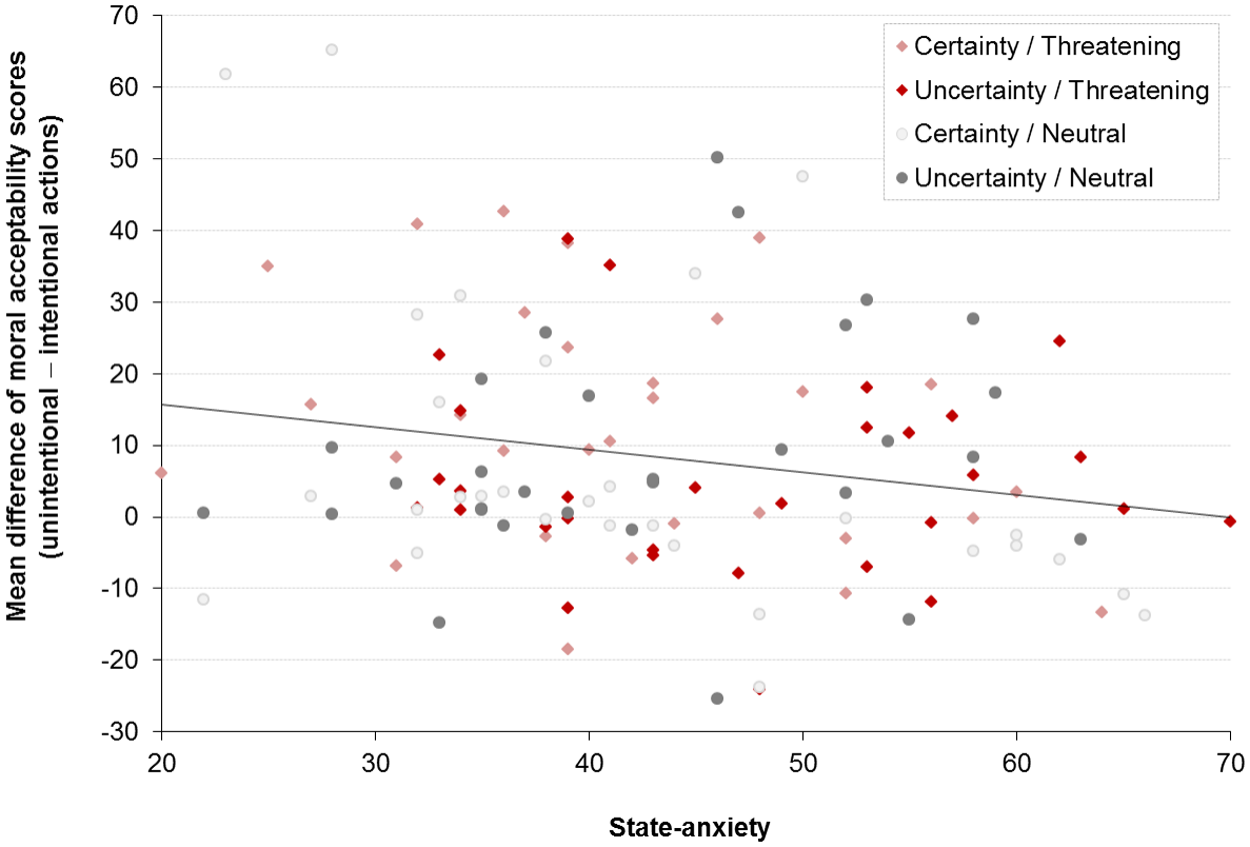
Relationship between state-anxiety and difference scores of moral acceptability (unintentional—intentional actions) (n = 120).

The ANOVAs conducted on the level of state and trait anxiety in function of the (un)certainty condition (certainty *vs*. uncertainty) and the context (threating *vs*. neutral) did not reveal any significant differences among the four groups (all *p* > .1). The detailed results are available as supplementary material (S2 Table). The simple regression analyses revealed that the mean level of state-anxiety reduced the difference between intentional and unintentional actions (Fig 4). The more anxious the individuals were, the less they presented differences in moral acceptability between intentional and unintentional actions, *F*(1, 118) = 5.09, R^2^ = .041, *β* = −.203, *p* = .026. Participant’s’ predicted perception of moral acceptability was equal to − .316(state-anxiety) + 22.06. The other regressions were not significant (all *p* > .1).

## Discussion

In a framed decision-making task with moral dilemma, we examined whether uncertainty stemming from unpredictable events and threatening contexts would influence the framing and personal intention effects on the moral acceptability of the choice. As required by the design of our experiment, we observed the well-known main effects of the framing and the personal intention on the moral acceptability scores. Such effects have often been observed in other works (e.g., ref. [7–9,19,33,43]) but with separate and distinct methodological designs: those for examining the framing effect [7,8,33,43] and those for examining the personal intention effect [9,19]. This constitutes a key argument in favor of the relevance of our new integrative paradigm to investigate framed decision-making with moral dilemmas.

### Uncertainty leads to the negation of the framing effect but does not influence moral judgments

With regards to our main hypotheses, we predicted and validated that uncertainty stemming from unpredictable events would lead to a negation of the framing effect applied to moral judgments. No difference in the moral acceptability of taking action was seen between the gain and loss framings for participants in the uncertainty conditions. This negation following uncertain events, regardless of its valence (negative or neutral), can be explained by appraisal theories. The identification of novelty, eliciting an orientation response towards a stimulus, precedes the valence appraisal [22]. Therefore, the exposure to unpredictable stimuli can lead to a heightened state of arousal and alertness [3,25,28] and follows a very similar pattern to that of uncertainty-associated emotions [3,25,44]. This would give rise to heightened systematic processing (system 2) and a reduced consideration of anticipatory emotions [21,23], which was exhibited by a longer RT in our data [17,45]. Conversely, since the framing effect comes from the processing of anticipatory emotions related to loss (system 1), such an activation typically leads to its negation [31]. In addition, uncertainty would be sufficient to elicit stress [28,46,47], which also involves heightened systematic processing (system 2) [29,33], and would lead to a similar negation of the framing effect [33].

In contrast, uncertainty did not influence either the moral acceptability (of taking action) or the effect of personal intention on this acceptability. Two explanations may help with this contradiction. First, despite their common tendency to reduce the processing of anticipatory emotions [6], there are key differences between the processing of moral judgments and the framing effect. Whereas many implicit processes underline the framing effect [31,48], moral judgments are rather characterized by conscious and deliberate features [6]. For example, judging an action as morally acceptable results from controlled rational concerns (system 2) that increase the acceptability of the tradeoff option [6,10] through an in-depth examination of the situation [18]. As the perception of uncertainty mainly relies on cognitive appraisals [25,44,49], this could explain why it exhibits a predictive value on the framing effect but not on moral judgments. Second, focusing on the action or the non-action in the presentation of the dilemma impacted the effect of emotions on moral acceptability in opposite manners [16]. Therefore, in our dilemma task, the lack of effect on personal intention might therefore be due to the consequences of simultaneously presenting both the taking action and the not taking action answers.

### Threatening context might affect moral judgments

We also predicted that the threatening context, compared to the neutral context, would lead to the negation of both framing and personal intention effects, which was not supported by our results. It is still important to note that the threatening context conditions respectively included the uncertainty and certainty modalities. Thus, the differential impact of the neutral and threatening contexts between-subjects conditions on the modalities of the framing and personal intention may have been too undermined to reach significance. This methodological and statistical consideration could account for the fact that we only obtained a trend toward a main effect of the context on the moral acceptability scores. More specifically, individuals in the threatening context condition tended to judge the actions as more morally acceptable than individuals in the neutral condition, which remains in agreement with the literature about the effect of emotions on moral dilemmas [6]. We still observed an inverse relationship between participants’ state-anxiety and the personal intention, in other words, the more anxious the participants were, the more the personal intention effect decreased. State-anxiety has often been considered as being strongly related to fear and stress responses [27]. Thus, when the anxiety response was implemented as a continuous independent variable, its relation with the decrease of the personal intention effect became more evident.

### Limitations

The present study has some limitations, which we delineate below. Firstly, females were overrepresented in our sample (111 for 9 males), and a gender effect could constitute a limit to our interpretations. With regard to the individuals’ reactivity to unpredictability, recent works have shown that gender had no influence on either individuals’ reactivity to unpredictability [25,26,49,50], nor on the framing effect [7,33,43,48,51]. Indeed, the largest literature reviews and meta-analyses did not report any established gender effect on the framing of loss and gain (e.g.,[52,53]). Only one review mentioned a possible gender effect, in which the authors clearly stated that it was impossible to conclude gender effects regarding the sensitivity to gain and loss frames [54]. Similarly, no specific gender effect has been reported for the personal intention in moral judgment [6,9,14–16,19,55–58]. Although males would tend to present slightly more utilitarian behaviors compared to females [59], a meta-analysis (N = 8778) showed that the effect sizes of such gender effect would be too small to account for a significant influence on moral judgments [60]. Finally, another large-scale study (N > 5000) [56] showed no gender effect, since males and females behaved equally with regard to the personal intention. Considering these elements, some key articles either mentioned only one gender (e.g., [16,33,61]) or did not even considered the gender (e.g., [14,40,57]). However, we caution readers that, while the literature did not account for any established gender effect on an individual’s reactivity to unpredictability (e.g., [25,26]), to the framing effect (e.g., [52,53]) or to personal intention in moral judgment (e.g., [56–58]), the possibility of a gender effect still remains, even if it would be very unlikely. We must therefore be cautious about the generalization of the results of our study given that they were mostly obtained from females. Potential applications of our results to a more general population should only be made after careful in situ re-testing, as it could be possible that males are less responsive to unpredictability and its associated negation of the framing effect. More precisely, with regards to the question of sampling limitation, we must specify that our sample was composed of western European individuals, recently considered as W.E.I.R.D (Western, educated, industrialized, rich and democratic), and represent only a portion of the world population [62]. We admit that this is a known issue that most studies cannot escape and that our study relies on the framing effect, which is among the most robust effects in decision-making, as first shown by Kahneman and Tversky in 1979 [63]. We also emphasize that our results warrant replication in other populations with different cultural characteristics (e.g., poorly educated individuals, eastern cultures, people at risk of volcanic hazards). In addition to improving the impact of our findings, it would also give crucial information regarding indeterminate cultural differences that may have been skipped in previous studies.

Second, the visual and auditory stimuli used in the induction task were not systemically congruent (e.g., no sound of a barking dog). This could lead to ambiguity, which has been associated with heightened amygdala activity, vigilance and attention [64]. However, ambiguity is also associated to unpredictability [25] and would have amplified the effect of this particular trait [50]. This is in fact what we observe when neutral facial expressions are used as control stimuli, since such a neutrality is not familiar and leads to ambiguity [65]. Thirdly, the levels of state anxiety did not differed significantly between groups, yet the mean scores were higher in the condition of uncertainty. This lack of significance could result from the subjective self-report method used to evaluate the state anxiety, which can lead to evaluation bias [66]. The cumulative use of another measure, such as skin conductance or heart-rate variability, should be explored in a replication of this study.

Finally, another explanation could also account for the relationship that we found between the anxiety response and the decrease of the personal intention effect. Emotion regulation strategies [67], including both cognitive reappraisal (reinterpreting the meaning of the situation through a different perspective, which reduces negative feelings) and emotional suppression (masking one’s actual emotional state), would alter the process of anticipatory emotions, thereby impacting decision-making (e.g., by increasing or decreasing risk aversion [68]) and the framing effect (e.g., by negating the framing effect in a risk-taking task [48]) [4,69]. However, to date, no study has investigated their after-effect on loss aversion. In our study, participants in the certainty condition may have adopted some of those strategies before the onset of the stimuli, which would have modified their emotional response and changed their decision-making. As we obtained strong results toward the negation of the framing effect following the exposure to uncertain events, future studies should investigate whether individuals tend to use a specific emotion regulation strategy when facing predictable and unpredictable threatening events. If one of these strategies could show its benefit to prevent an inappropriate increase of the systematic processes (system 2) following exposure to uncertainty, the way the situation is presented (e.g., framing, intentionality) would bias less moral decision-making and would allow individuals to better cope with threatening and unpredictable events.

### Conclusion

In a decision-making task with moral dilemma, the perception of uncertainty stemming from unpredictable events would result in the negation of the framing effect, probably due to a heightened systematic processing (system 2) and the resulting reduction of the anticipatory emotional processing. This stresses the need to devise a solution for scientists and local populations to deal properly with uncertainty, especially in cases of impending natural disasters. We reviewed the possibility of cognitive emotion regulation during uncertain events, which still need to be evaluated. Another intuitive solution would be to induce certainty in exposed population, but it is not feasible since it could result in false alarms and a low credibility of the relevant authorities. Further lines of research should instead focus on how to use cognitive strategies to regulate emotions for appropriate moral decision-making in cases of crisis events.

## Supporting information

S1 Video Video extract of the breakout game. (MP4)

S2 Video Video extract of the moral dilemma task. (MP4)

S1 Data Experimental data. (XLSX)

S1 Table Results from the repeated-measures ANOVA and descriptive statistics conducted on the moral acceptability scores. (DOCX)

S2 Table Results from the ANOVA and descriptive statistics conducted on the levels of state and trait anxiety. (DOCX)
