# Supplementary material for: Influence of uncertainty on framed decision-making with moral dilemma
Source: PLoS One. 2018 May 30;13(5):e0197923. doi: 10.1371/journal.pone.0197923 (PMC5976155; doi:10.1371/journal.pone.0197923)
Supplement: S1 Table — (DOCX) [file pone.0197923.s004.docx]

**S1 Table. Results from the repeated-measures ANOVA and descriptive statistics conducted on the moral acceptability scores**

| **MAIN EFFECTS** | | | | |  | | |
| --- | --- | --- | --- | --- | --- | --- | --- |
|  | ***F*-value** | ***p-value*** | **η_p_²** | **Condition** | **M ± SD** | | |
| **Framing** | 11.93 | .001*** | .093 | Gain | 51.89 ± 22.15 | | |
|  |  |  |  | Loss | 47.97 ± 22.56 | | |
| **Personal intention** | 27.63 | < .001*** | .19 | Intentional | 45.76 ± 23.58 | | |
|  |  |  |  | Unintentional | 54.09 ± 22.58 | | |
| **(Un)certainty** | 0.017 | .896 | .0002 | Certainty | 49.67 ± 20.15 | | |
|  |  |  |  | Uncertainty | 50.18 ± 22.75 | | |
| **Context** | 3.49 | .064 ^t^ | .029 | Threatening | 53.56 ± 21.42 | | |
|  |  |  |  | Neutral | 46.30 ± 20.94 | | |
| **INTERACTION EFFECTS** | | | | |  | | |
|  | ***F*-value** | ***p-value*** | **η_p_²** | **Condition** | **M ± SD** | | |
| **(Un)certainty * Framing** | 9.62 | .002** | .077 |  |  | |  |
| *In certainty condition* | 21.48 | <.001*** | .16 | Gain  Loss | 53.40 ± 21.45  45.95 ± 21.35 | |  |
| *In uncertainty condition* | 0.062 | .804 | .001 | Gain  Loss | 50.38 ± 22.91  49.98 ± 23.71 | |  |
| *In gain condition* | 0.57 | .453 | .005 | Certainty  Uncertainty | 53.40 ± 21.45  50.38 ± 22.91 | |  |
| *In loss condition* | 0.97 | .326 | .008 | Certainty  Uncertainty | 45.95 ± 21.35  49.98 ± 23.71 | |  |
| **Context * Framing** | 1.07 | .304 | .009 |  |  | |  |
| *In threatening context* |  |  |  | Gain  Loss | 56.11 ± 22.88  51.01 ± 22.44 | |  |
| *In neutral context* |  |  |  | Gain  Loss | 47.67 ± 20.73  44.92 ± 22.45 | |  |
|  | ***F*-value** | ***p-value*** | **η_p_²** | **Condition** | | **M ± SD** | |
| **(Un)certainty * Personal intention** | .78 | .379 | .007 |  |  | |  |
| *In certainty condition* |  |  |  | Intentional  Unintentional | 44.81 ± 23.04  54.54 ± 21.70 | |  |
| *In uncertainty condition* |  |  |  | Intentional  Unintentional | 46.72 ± 24.27  53.64 ± 23.61 | |  |
| **Context * Personal intention** | .019 | .889 | .0002 |  |  | |  |
| *In threatening context* |  |  |  | Intentional  Unintentional | 49.28 ± 23.58  57.83 ± 21.99 | |  |
| *In neutral context* |  |  |  | Intentional  Unintentional | 42.24 ± 23.25  50.35 ± 22.73 | |  |
| **Framing * Personal intention** | .13 | .721 | .001 |  |  | |  |
| *In gain condition* |  |  |  | Intentional  Unintentional | 47.54 ± 25.18  56.24 ± 24.00 | |  |
| *In loss condition* |  |  |  | Intentional  Unintentional | 43.99 ± 24.94  51.94 ± 24.43 | |  |
| **(Un)certainty * Context * Framing** | 1.25 | .267 | .011 |  |  | |  |
| *In certainty condition* |  |  |  |  |  | |  |
| *In threatening context* |  |  |  | Gain  Loss | 56.37 ± 22.55  46.48 ± 18.45 | |  |
| *In neutral context* |  |  |  | Gain  Loss | 50.42 ± 20.23  45.42 ± 24.22 | |  |
| *In uncertainty condition* |  |  |  |  |  | |  |
| *In threatening context* |  |  |  | Gain  Loss | 55.84 ± 23.59  55.53 ± 25.33 | |  |
| *In neutral context* |  |  |  | Gain  Loss | 44.92 ± 21.21  44.43 ± 20.92 | |  |
|  | ***F*-value** | ***p-value*** | **η_p_²** | **Condition** | | **M ± SD** | |
| **(Un)certainty * Context * Personal intention** | 1.79 | .183 | .015 |  |  | |  |
| *In certainty condition* |  |  |  |  |  | |  |
| *In threatening context* |  |  |  | Intentional  Unintentional | 45.39 ± 21.75  57.46 ± 19.27 | |  |
| *In neutral context* |  |  |  | Intentional  Unintentional | 44.23 ± 24.62  51.61 ± 23.86 | |  |
| *In uncertainty condition* |  |  |  |  |  | |  |
| *In threatening context* |  |  |  | Intentional  Unintentional | 53.17 ± 25.03  58.2 ± 24.75 | |  |
| *In neutral context* |  |  |  | Intentional  Unintentional | 40.26 ± 22.05  49.09 ± 21.87 | |  |
| **(Un)certainty * Framing * Personal intention** | .46 | .5 | .004 |  |  | |  |
| *In certainty condition* |  |  |  |  |  | |  |
| *In gain condition* |  |  |  | Intentional  Unintentional | 47.99 ± 25.85  58.80 ± 22.71 | |  |
| *In loss condition* |  |  |  | Intentional  Unintentional | 41.63 ± 24.36  50.27 ± 24.22 | |  |
| *In uncertainty condition* |  |  |  |  |  | |  |
| *In gain condition* |  |  |  | Intentional  Unintentional | 47.08 ± 24.71  53.68 ± 25.16 | |  |
| *In loss condition* |  |  |  | Intentional  Unintentional | 46.35 ± 25.49  53.61 ± 24.73è | |  |
| **Context * Framing * Personal intention** | 1.245 | .267 | .011 |  |  | |  |
| *In threatening context* |  |  |  |  |  | |  |
| *In gain condition* |  |  |  | Intentional  Unintentional | 52.23 ± 25.05  59.98 ± 23.87 | |  |
| *In loss condition* |  |  |  | Intentional  Unintentional | 46.34 ± 25.50  55.68 ± 23.61 | |  |
|  | ***F*-value** | ***p-value*** | **η_p_²** | **Condition** | | **M ± SD** | |
| *In neutral context* |  |  |  |  |  | |  |
| *In gain condition* |  |  |  | Intentional  Unintentional | 42.85 ± 24.63  52.49 ± 23.74 | |  |
| *In loss condition* |  |  |  | Intentional  Unintentional | 41.64 ± 24.35  48.21 ± 24.86 | |  |
| **(Un)certainty * Context * Framing* Personal intention** | .416 | .52 | .004 |  |  | |  |
| *In certainty condition* |  |  |  |  |  | |  |
| *In threatening context* |  |  |  |  |  | |  |
| *In gain condition* |  |  |  | Intentional  Unintentional | 50.04 ± 26.56  62.70 ± 22.11 | |  |
| *In loss condition* |  |  |  | Intentional  Unintentional | 40.74 ± 21.45  52.22 ± 21.88 | |  |
| *In neutral context* |  |  |  |  |  | |  |
| *In gain condition* |  |  |  | Intentional  Unintentional | 45.94 ± 25.40  54.90 ± 23.01 | |  |
| *In loss condition* |  |  |  | Intentional  Unintentional | 42.51 ± 27.30  48.32 ± 26.59 | |  |
| *In uncertainty condition* |  |  |  |  |  | |  |
| *In threatening context* |  |  |  |  |  | |  |
| *In gain condition* |  |  |  | Intentional  Unintentional | 54.41 ± 23.69  57.27 ± 25.61 | |  |
| *In loss condition* |  |  |  | Intentional  Unintentional | 51.93 ± 28.24  59.13 ± 25.12 | |  |
| *In neutral context* |  |  |  |  |  | |  |
| *In gain condition* |  |  |  | Intentional  Unintentional | 39.76 ± 23.87  50.09 ± 24.60 | |  |
| *In loss condition* |  |  |  | Intentional  Unintentional | 40.77 ± 21.44  48.09 ± 23.45 | |  |

Notes: η_p_²: partial eta squared; M ± SD: mean ± standard deviation; simple effects are reported to decompose the significant interaction effect only.
