# Supplementary material for: Influence of uncertainty on framed decision-making with moral dilemma
Source: PLoS One. 2018 May 30;13(5):e0197923. doi: 10.1371/journal.pone.0197923 (PMC5976155; doi:10.1371/journal.pone.0197923)
Supplement: S2 Table — (DOCX) [file pone.0197923.s005.docx]

**S2 Table. Results from the ANOVA and descriptive statistics conducted on the level of state and trait anxiety**

**TRAIT ANXIETY**

| **MAIN EFFECTS** | | | | |  | |
| --- | --- | --- | --- | --- | --- | --- |
|  | ***F*-value** | ***p-value*** | **η_p_²** | **Condition** | **M ± SD** | |
| **(Un)certainty** | 0.057 | .811 | .0005 | Certainty | 45.10 ± 7.32 | |
|  |  |  |  | Uncertainty | 45.40 ± 6.31 | |
| **Context** | 0.918 | .34 | .008 | Threatening | 44.65 ± 6.18 | |
|  |  |  |  | Neutral | 45.85 ± 7.39 | |
| **INTERACTION EFFECTS** | | | | |  | |
|  | ***F*-value** | ***p-value*** | **η_p_²** | **Condition** | **M ± SD** | |
| **(Un)certainty * Context** | 0.23 | .633 | .002 |  |  |  |
| *In certainty condition* |  |  |  | Threatening  Neutral | 44.80 ± 6.69  45.40 ± 8.01 |  |
| *In uncertainty condition* |  |  |  | Threatening  Neutral | 44.50 ± 5.73  46.30 ± 6.82 |  |

**STATE ANXIETY**

| **MAIN EFFECTS** | | | | |  | |
| --- | --- | --- | --- | --- | --- | --- |
|  | ***F*-value** | ***p-value*** | **η_p_²** | **Condition** | **M ± SD** | |
| **(Un)certainty** | 2.32 | .131 | .02 | Certainty | 41.97 ± 11.38 | |
|  |  |  |  | Uncertainty | 45.05 ± 10.81 | |
| **Context** | 0.718 | .399 | .006 | Threatening | 44.37 ± 10.91 | |
|  |  |  |  | Neutral | 42.65 ± 11.43 | |
| **INTERACTION EFFECTS** | | | | |  | |
|  | ***F*-value** | ***p-value*** | **η_p_²** | **Condition** | **M ± SD** | |
| **(Un)certainty * Context** | 1.23 | .269 | .011 |  |  |  |
| *In certainty condition* |  |  |  | Threatening  Neutral | 41.70 ± 10.45  42.23 ± 12.42 |  |
| *In uncertainty condition* |  |  |  | Threatening  Neutral | 47.03 ± 10.87  43.07 ± 10.55 |  |

Notes: η_p_²: partial eta squared; M ± SD: mean ± standard deviation; simple effects are reported to decompose the significant interaction effect only.
